# Clinical and laboratory features of different types of cancer-associated thrombosis

**DOI:** 10.7150/jca.89231

**Published:** 2023-10-30

**Authors:** Yanhong Liu, Rufei Ma, Yue Li, Lan Gao

**Affiliations:** Department of Laboratory Medicine, Henan Provincial People's Hospital, People's Hospital of Zhengzhou University, People's Hospital of Henan University, Zhengzhou, Henan, 450003, China.

**Keywords:** Venous thrombosis, Gastrointestinal cancer, Lung cancer, Gynecological cancer

## Abstract

**Background:** Patients with cancer showed a high incidence of venous thromboembolism (VTE) with a poor prognosis. The risk factors for VTE in different types of cancers may differ.

**Methods:** The clinical features and laboratory test results of cancer patients with VTE in Henan Provincial People's Hospital from 2014 to 2020 were evaluated and compared.

**Results:** Among the eligible patients, gastrointestinal cancer (GI cancer), lung cancer and gynecological cancer accounted for the top three. This study included 49 patients with GI cancer, 31 with lung cancer and 31 with gynecological cancer. The proportion of patients who underwent surgery in GI cancer or gynecological cancer group was significantly higher than that for lung cancer (69.4% and 80.6% vs 12.9%, both *P*<0.001). Red blood cell (RBC) and hemoglobin (HGB) levels were lower in the gynecological cancer group than that in the lung cancer group (P = 0.014 and 0.029, respectively), while red cell distribution width (RDW) was higher in the GI cancer group than that in the lung cancer group and gynecological cancer group (*P* = 0.047 and 0.010, respectively). Prothrom bin time (PT) was shorter in the gynecological cancer group than that in the GI and lung cancer group (*P* = 0.003 and* P* = 0.002, respectively). The activated partial thromboplastin time (APTT) in the lung cancer group was longer than that in the GI and gynecological cancer group (*P* = 0.029 and 0.003, respectively). There was no difference in LOS and successful treatment rate among the groups. However, the VTE cure rate in the gynecological cancer group is higher than that in the GI cancer group (90.3% vs 61.2%, *P* = 0.005). The probability of continuing to take anticoagulants after discharge in the gynecological cancer group is lower than that in the GI and lung cancer groups (6.5% vs 30.6% and 32.3%, *P* = 0.011 and 0.022 respectively).

**Conclusion:** VTE risk factors of different types of cancers and laboratory test results were not exactly the same. Thrombosis prevention and treatment should be implemented according to the characteristics of the different types of cancer.

## 1. Introduction

Cancer is an independent risk factor for venous thromboembolism (VTE). Patients with cancer have a 4-6.5-fold increased risk of developing VTE compared with other patients 1. Cancer-associated thrombosis (CAT) causes a 3-fold increase in mortality and deterioration of quality of life 2. Although the association between cancer and VTE is well recognized, information on CAT in different types of cancers is currently scarce. Some studies have found that the prevalence of CAT varied considerably according to the type of cancer 3, 4. Therefore, we believe that the characteristics of CAT in different types of cancer are not identical. Previous research has only focused on a single type of cancer, and has devoted to molecular mechanisms or the effectiveness of anticoagulant therapy. However, few studies have compared the clinical features and laboratory test results of different CAT. VTE prevention and treatment are more complex for patients with cancer. Understanding of the characteristics of different CAT may facilitate the development of precision medicine. In the current analysis, we collected clinical data and laboratory test results and discussed the characteristics of different CAT.

## 2. Methods

### 2.1 Study design and population

From April 2014 to April 2020, a total of 80963 patients with cancer were admitted to Henan Provincial People's Hospital. VTE occurred in 1747 patients (2.16%). Patients with other VTE risks such as pregnancy and fractures were excluded as it cannot be determined whether their VTE is caused by cancer. Finally, 120 patients met the inclusion criteria. All diagnoses were confirmed according to the most current guidelines 5, 6. Demographic data and cancer details including age, comorbidities, cancer type, anticoagulant use, and applied treatment (chemotherapy, radiotherapy, surgery and implementation of central venous catheter) were collected. Patients are difficult to completely cure as all participants are cancer patients. We defined successful treatment as if the condition was improved and the discharge criteria were met. Death and giving up as unsuccessful treatment. The length of stay (LOS), outcome (successful or unsuccessful), whether VTE was cured before discharge and whether continued anticoagulation was required after discharge were recorded. This study was approved by Human Research Ethics Committee of Henan Provincial People's Hospital.

### 2.2 Specimen collection and measurements

Venous blood samples were collected from each participant into two vacuum tubes. Blood samples for complete blood count were drawn into tubes containing K_2_ EDTA and analyzed using Sysmex XN9100. Blood samples for coagulation assays were drawn into tubes containing 3.2% sodium citrate as anticoagulant and were centrifuged at 1,500 × g for 10 minutes to separate the plasma. Coagulation assays were performed using a Sysmex CS 5100 automatic coagulation analyzer.

### 2.3 Statistical analysis

Statistical analyses were carried out using SPSS 25. The Shapiro-Wilk test was used to determine whether continuous variables (Age, BMI, RBC, WBC, PLT, HGB, RDW, PDW, PT, APTT, Fib, TT, DD, LOS) had a normal distribution. Normally distributed data were expressed as the means ± standard deviations. One-way ANOVA was performed to compare the difference among the three group (*P*<0.05) and pairwise comparison (LSD test) was used to compare differences between each two group. Non-normally distributed data were reported as medians (interquartile range) and compared using Kruskal-Wallis test, then pairwise comparisons were made to determine which two groups have difference if *P*<0.05. Categorical variables (gender, smoking status, comorbidities, VTE type, time of VTE, VTE related factors, VTE treatment, successful treatment, VTE cured, anticoagulation after discharge) were described by percentages and compared using chi-square test or Fisher's test (*P*<0.05).

## 3. Results

### 3.1 Clinical characteristics of participants

**Figure 1:**
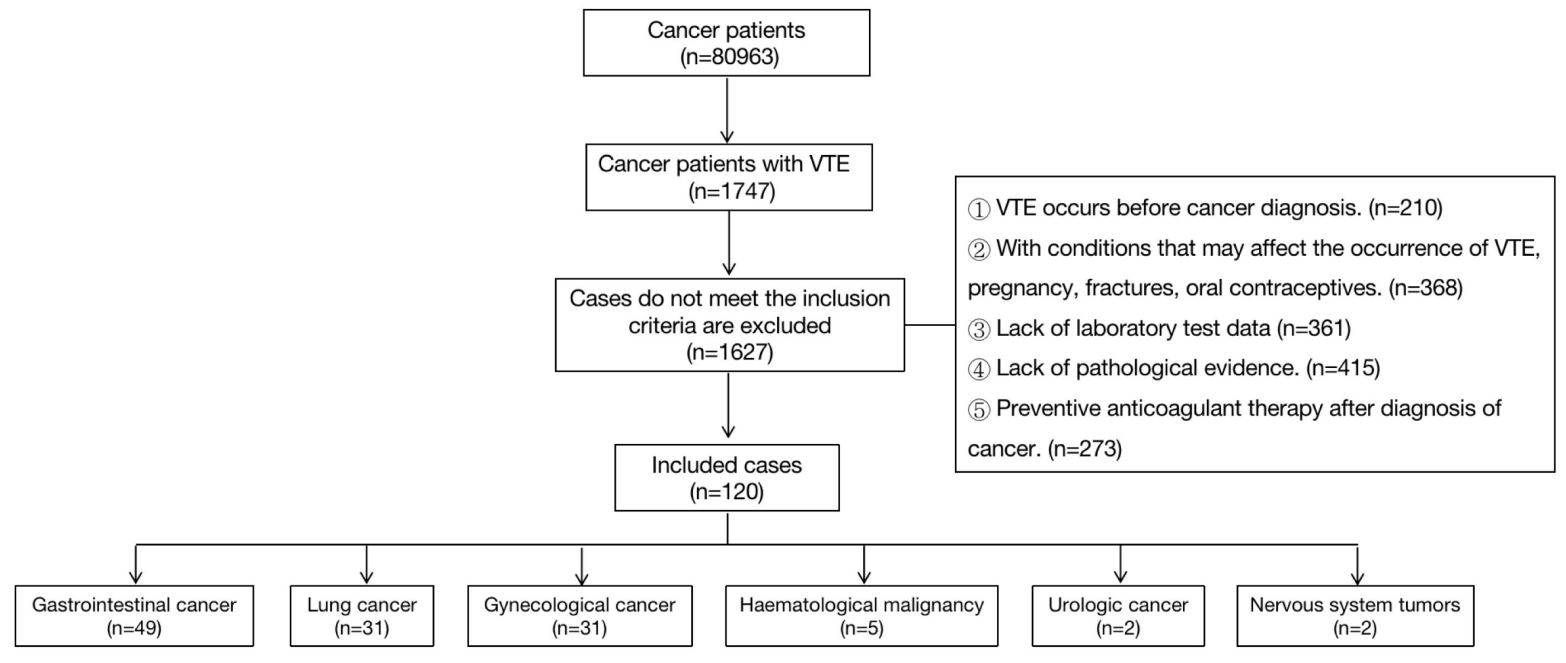
Diagram of patient selection process.

Among the 120 cancer patients with VTE, the top three cancer types were gastrointestinal cancer (49 patients, 40.8%), lung cancer (31 patients, 25.8%), and gynecological cancer (31 patients, 25.8%). Others included tumor of the urinary system, hematological tumors and nervous system tumors. (nine patients, 7.5%). Patients with gastrointestinal cancer (GI cancer) included 32 cases of colorectal cancer, seven cases of liver cancer, four cases of gastric cancer, three cases of pancreatic cancer, two cases of biliary system tumors, and one case of esophageal cancer. Gynecological cancers included 13 cases of ovarian cancer, five cases of endometrial cancer, seven cases of cervical cancer and six cases of breast cancer (Figure 1). Patients with adenocarcinoma in the GI, lung and gynecological cancers groups accounted for 63.3%, 64.5%, and 41.9%, respectively. The baseline demographic and clinical characteristics of the patients are shown in Table 1. Patients in the GI cancers group were older than those in the gynecological cancers group. There were no significant differences in other baseline demographic characteristics among the groups.

### 3.2 The characteristics of CAT

There were no significant differences in the type and time of occurrence of VTE among the three groups. However, the GI and gynecological cancer groups had a significantly higher number of surgical patients than that of the lung cancer group. The results are presented in Table 2.

### 3.3 Laboratory test results

Red blood cell (RBC) and hemoglobin (HGB) levels in the gynecological cancer group were significantly lower than those in the lung cancer group. The red cell distribution width (RDW) in GI cancer group was significantly higher than that in the lung cancer and gynecological cancer group. The prothrombin time (PT) in the gynecological cancer group was significantly lower than that in GI and lung cancer group. Activated partial thromboplastin time (APTT) in the lung cancer group was significantly higher than that in the GI cancer group and gynecological cancer group (Table 3).

### 3.4 Prognosis

There was no difference in LOS and successful treatment rate among the groups. However, the VTE cure rate in the gynecological cancer group is higher than that in the GI cancer group. The probability of continuing to take anticoagulants after discharge in the gynecological cancer group is lower than that in the lung and GI cancer groups.

## 4. Discussion

CAT is common in patients with cancer and increases morbidity and mortality. According to our study, the incidence of CAT in the past 6 years was 2.16% and most CAT cases occurred in the first 6 months after the diagnosis of cancer which is consistent with previous studies 7-9. This may be related to the higher cancer burden at the time of diagnosis, which decreases with effective therapy and more attention should be paid during this period. Previous studies have reported that patients with pancreatic, ovarian, brain, stomach, gynecologic, and hematologic cancer are at high VTE rate, and colon and lung are intermediate 4. In our population, gastrointestinal, lung and gynecological cancer accounted for the highest proportion. This is also related to the incidence rate of different types of cancer.

The risk of CAT depends on multiple factors including patient, cancer and treatment related factors. We found that the number of patients who underwent surgical treatment in the GI and gynecological cancer group were higher than those in the lung cancer group. Previous clinical studies have linked surgical treatment to a significant risk of VTE due to the placement of long-term catheters and postoperative brake. However, some researchers contend that surgical treatment can reduce the risk of CAT in cancer patients, which may be related to the decreased in procoagulants secreted by tumor cells as the tumor was removed and the management of perioperative anticoagulation. However, whether surgery increases the risk of VTE in cancer patients remains unclear.

Some studies have suggested that there may be cancer type-specific pathways of CAT 4. For example, leukocytosis is most frequently observed in patients with lung or colorectal cancer. Thrombocytosis was often observed in patients with ovarian cancer. However, our data shows that white blood count (WBC) and platelet (PLT) were not significantly different among the three groups, which may be due to the small sample size. Further studies are required to confirm this hypothesis.

Cancer patients are not only high-risk groups for CAT, but also high-risk groups for bleeding. In our study, three patients in the GI cancer group did not receive any anticoagulant therapy because of their bleeding or the risk of bleeding. Patients with a high risk of bleeding are not suitable for using anticoagulants. In these patients, how to treat VTE becomes a difficult problem. In addition, the anticoagulant strategies used by the patients in the different groups were not the same. The number of patients in the gynecological cancer group who received thrombolytic therapy was higher than that of the lung cancer group. Balancing thrombosis and bleeding in cancer patients has become an urgent problem. Individualized prevention and treatment measures for CAT should be implemented.

Paitan 10 et al. found that more than 50% of the patients with GI or gynecological cancer were diagnosed with anemia. Our data showed that RBC and HGB levels in the gynecological cancer group were lower than those in the lung cancer group, which may be due to sex differences and the different incidences of anemia in different cancers. Most routine laboratory parameters in participants were within the normal range except for D-dimer (DD). The risk of VTE according to this reference range might be overestimated because DD in cancer patients' levels may be elevated even in the absence of VTE 11. Clinical studies have shown that DD levels vary among different cancers. DD levels in patients with gastrointestinal cancer are significantly higher than those in patients with breast cancer 12. To identify CAT early, specific DD ranges for different cancer types should be established and continuously monitored. Routine laboratory parameters cannot meet the requirements for early diagnosis of CAT. Perhaps we can evaluate the risk of CAT through genetic testing. For example, mutations in K-Ras in colon and lung cancers are associated with an increased risk of VTE, as is JAK2 V617F in myeloproliferative cancer 13, 14.

VTE can lead to longer LOS for cancer patients 1, but there are no studies comparing the LOS in patients with VTE with different cancer types. Our data shows that there is no significant difference in LOS among VTE patients with GI, lung and gynecological cancers which need to be verified in a larger sample population. After a period of treatment, the symptoms of VTE for some patients were relieved, but the imaging examination is still positive. There were more such patients in the GI cancer group and fewer in the gynecological cancer group, however, our sample size was small and the age of the gynecological group was younger, so further verification was needed. Some patients need to continue long-term anticoagulation after discharge 15. Our study found that this number varies among different types of cancers. The follow-up work was only conducted before discharge due to poor patient compliance.

## Figures and Tables

**Table 1:** Baseline clinical characteristics

Characteristic	GI cancer (n = 49)	Lung cancer (n = 31)	Gynecological cancer (n = 31)	*P* Value
Age, mean (SD)	63.0±12.1	57.1±12.2	55.2±11.0	0.009*
Male/Female, n	21/28	10/21	0 /31	0.360
BMI, mean (SD)	23.7±3.4	23.8±2.7	25.8±3.8	0.060
Smoking status, n (%)				
Former	3 (6.1)	3 (9.7)	0	0.821
Current	16 (32.7)	9 (29.0)	0	
No	30 (61.2)	19 (61.3)	31 (100)	
Comorbidities, n (%)				
Hypertension	14 (28.6)	4 (12.9)	9 (29.0)	0.264
Cardiovascular disease	9 (18.4)	2 (6.5)	1 (3.2)	0.079
Diabetes	8 (16.3)	1 (3.2)	4 (12.9)	0.202
COPD	0	3 (9.7)	0	0.050

Notes: Sex and smoking history only were compared between GI and lung cancer group. *P<0.05 and the difference was between GI and gynecological cancer group.

**Table 2:** Comparison of the characteristics of different CAT types

CAT features	GI cancer (n = 49)	Lung cancer (n = 31)	Gynecological cancer (n = 31)	*P* Value
VTE type				
DVT, n (%)	38 (77.6)	16 (51.6)	22 (71.0)	0.095
PTE, n (%)	9 (18.4)	9 (29.0)	5 (16.1)	
PTE+DVT, n (%)	2 (4.0)	6 (19.4)	4 (12.9)	
Time of VTE diagnosis				
0-6 months after cancer diagnosis, n (%)	37 (75.5)	25 (80.6)	23 (74.2)	0.505
6-12 months after cancer diagnosis, n (%)	3 (6.1)	2 (6.5)	0	
More than 12 months after cancer diagnosis, n (%)	9 (18.4)	4 (12.9)	8 (25.8)	
VTE related factors				
Immobilization, n (%)	3 (6.1)	1 (3.2)	3 (9.7)	0.703
Chemotherapy, n (%)	17 (34.7)	18 (58.1)	14 (45.2)	0.133
Surgery, n (%)	34 (69.4)	4 (12.9)	25 (80.6)	<0.001*
Central venous catheter, n (%)	7 (14.3)	3 (9.7)	0	0.072
VTE treatment				
Thrombolytic therapy, n (%)	4 (8.2)	0	2 (6.5)	0.196
Anticoagulant, n (%)	31(62.3)	25 (80.6)	16 (51.6)	
IVC filter, n (%)	11 (22.4)	5 (16.1)	12 (38.7)	
Untreated, n (%)	3 (6.1)	1 (3.2)	1 (3.2)	

Notes: *P<0.05 and pairwise comparison showed there was a significant difference between GI and lung cancer group, and there was a significant difference between gynecological and lung cancer group.

**Table 3:** Comparison of laboratory test results

Parameters	GI cancer (n = 49)	Lung cancer (n = 31)	Gynecological cancer (n = 31)	Reference range	*P* Value
RBC^c^, ×10^12^/L	3.74 ± 0.51	4.0 ± 0.76	3.53 ± 0.47	4.3-5.8	0.017*
WBC, ×10^9^/L	6.45 (4.21, 8.48)	7.40 (5.10, 9.90)	6.96 ±3.08	3.5-9.5	0.610
PLT, ×10^9^/L	208.90 ± 85.47	228.73 ± 95.76	227.94 ± 97.61	125-350	0.575
HGB^c^, g/L	107.46 ± 19.65	117.98±21.87	105.16 ± 14.38	130-175	0.022*
RDW^ab^, %	14.6 (13.8, 16.7)	13.6 (12.7, 15.1)	13.3 (12.4, 15.5)	10-15	0.005*
PDW, fL	13.0 (10.8, 14.75)	13.32 (11.0, 4.6)	12.0 (10.5, 14.0)	10-18	0.391
PT^bc^, s	14.0 (12.95, 17.19)	15.7 (12.10, 21.6)	12.97±1.78	11-17	0.001*
APTT^ac^, s	36.3 ± 5.83	40.9 (35.12, 4.9)	34.82 ± 4.87	28-43.5	0.003*
Fib, g/L	3.53 (2.77, 4.46)	3.89 (2.73, 5.23)	4.19 ± 1.32	2.0-4.0	0.220
TT, s	15.6 (15.0, 17.3)	16.7 (14.3, 17.91)	16.68 ± 2.27	14-21	0.325
DD, ug/ml	2.84 (1.67, 5.28)	2.76 (0.87, 4.30)	3.96 (1.74, 4.7)	0-0.5	0.322

Notes: *P<0.05. Pairwise comparison, a: there was a significant difference between GI and lung cancer group, b: there was a significant difference between GI and gynecological cancer group, c: there was a significant difference between lung and gynecological cancer group.

**Table 4:** Comparison of the prognosis of the three groups.

Prognosis	GI cancer (n = 49)	Lung cancer (n = 31)	Gynecological cancer (n = 31)	*P* Value
LOS	13.0 (9.5, 17.5)	15.0 (12.0, 17.0)	14.0 (10.0, 15.0)	0.452
Successful treatment, n (%)	35 (71.4)	23 (74.2)	28 (90.3)	0.126
VTE cured^b^, n (%)	30 (61.2)	24 (77.4)	28 (90.3)	0.014*
Anticoagulation after discharge^bc^, n (%)	15 (30.6)	10 (32.3)	2 (6.5)	0.024*

Notes: *P<0.05. Pairwise comparison, a: there was a significant difference between GI and lung cancer group. b: there was a significant difference between GI and gynecological cancer group, c: there was a significant difference between lung and gynecological cancer group.
